# A Pantheoretical Framework to Optimize Adherence to Healthy Lifestyle Behaviors and Medication Adherence: The Use of Personalized Approaches to Overcome Barriers and Optimize Facilitators to Achieve Adherence

**DOI:** 10.2196/16429

**Published:** 2020-06-24

**Authors:** Azizi Seixas, Colleen Connors, Alicia Chung, Tiffany Donley, Girardin Jean-Louis

**Affiliations:** 1 NYU Grossman School of Medicine New York, NY United States; 2 Georgetown University Georgetown, NY United States

**Keywords:** adherence, mHealth, management, chronic diseases, prevention, technology

## Abstract

Patient nonadherence to healthy lifestyle behaviors and medical treatments (like medication adherence) accounts for a significant portion of chronic disease burden. Despite the plethora of behavioral interventions to overcome key modifiable/nonmodifiable barriers and enable facilitators to adherence, short- and long-term adherence to healthy lifestyle behaviors and medical treatments is still poor. To optimize adherence, we aimed to provide a novel mobile health solution steeped in precision and personalized population health and a pantheoretical approach that increases the likelihood of adherence. We have described the stages of a pantheoretical approach utilizing tailoring, clustering/profiling, personalizing, and optimizing interventions/strategies to obtain adherence and highlight the minimal engineering needed to build such a solution.

## Introduction

### Background

Primary prevention *vis-à-vis* healthy lifestyle behaviors (healthy diet, nutrition, physical activity, sleep, and stress management) and management of chronic health conditions *vis-à-vis* pharmacological strategies significantly: (1) reduce the economic and medical burden of chronic diseases (eg, cardiometabolic disease and cancer) and corollary risk factors and (2) engender and improve positive health outcomes [1-8]. Poor adherence, the degree to which an individual does not follow pharmaceutical or lifestyle advice or regimen about primary prevention and management of chronic health conditions (such as lifestyle health behaviors and medications) [1] has significant economic and health consequences, resulting in greater health care expenditures, multiple morbidities, and deaths [9-11]. The burgeoning use of mobile technologies to deliver health, lifestyle and wellness interventions has shown initial signs of improving adherence to primary prevention and management of chronic health conditions [12-14]. However, the full potential of achieving optimized levels of adherence are thwarted by a wide range of sociodemographic, psychosocial, behavioral, and system-level barriers and the lack of personalized medicine and a precision population health approach—approaches that provide insight about the etiology of disease and health and promote customized, adaptive and just-in-time interventions based on biological/individual (eg, genes, biomarkers, circadian profile), lifestyle/behavioral (diet, physical activity, sleep, and stress management), and environmental/contextual (household, neighborhood, and cultural) factors. The purpose of this study was to explore (1) modifiable and nonmodifiable barriers and facilitators of adherence to primary prevention and management of chronic health conditions, especially in mobile health (mHealth) solutions; (2) a precision and personalized population health framework that overcomes barriers and enables facilitators to adherence in primary prevention and management of chronic health, noncommunicable, and communicable health conditions; and (3) how to implement a precision and personalized population health approach in mHealth/digital health solutions.

### Determinants of Health Adherence

Adherence to primary prevention and management of chronic health conditions in medicine and health is described as the degree to which an individual’s health and lifestyle behaviors are consistent with health recommendations [15,16]. Adherence can be influenced by (1) patient-related, (2) condition-related, (3) social and economic, (4) therapy-related, and (5) health system factors [17,18], which can be clustered into nonmodifiable (eg, race, gender, age) and modifiable (eg, health literacy, social support, and stress levels) factors. Distinguishing nonmodifiable and modifiable factors provides insights into how to modulate the effects of these barriers to improve adherence. For example, interventions based on and aimed at addressing nonmodifiable factors may not provide the best opportunities to improve and optimize adherence. Conversely, interventions based on and aimed at addressing modifiable factors may provide better opportunities to improve and optimize adherence to primary prevention and management of chronic health condition strategies.

#### Nonmodifiable Determinants of Adherence

Nonmodifiable factors are generally immutable and/or recalcitrant to change, which include sociodemographic (eg, age, race/ethnicity, sex) and psychosocial and behavioral (socioeconomic status [SES]) factors that individuals have little to no control over. Traditionally, adherence to primary prevention and management of chronic health condition solutions mistakenly target nonmodifiable factors to alter and increase adherence behaviors. Such an assumption would yield flawed assertions such as your race or sex determines your level of adherence to therapies and healthy behaviors. This should not be confused with acknowledging the likelihood that someone’s race or sex may be an important factor in their adherence behaviors. The distinguishing factor between the two approaches is that the former uses nonmodifiable factors to develop interventions that target subgroups, whereas the latter uses nonmodifiable factors to tailor interventions for subgroups of individuals that share common lived experiences (eg, neighborhood). For example, instead of targeting blacks, we can tailor interventions for low-income blacks living in midsized urban neighborhoods.

We acknowledge that there are rare times when nonmodifiable factors are the root cause of nonadherence behaviors, such as race-specific adverse medication side effects and suboptimal response to medications by certain racial/ethnic groups (eg, ACE inhibitors). Despite this, we argue that the fundamental premise of interventions aimed at increasing adherence behaviors is to modify factors that facilitate or impede adherence and to do this exclusively with nonmodifiable factors (eg, race, family history, and SES) is impossible. Instead, the process of tailoring interventions around nonmodifiable factors, whereby contents and activities of an intervention are geared toward cohorts of people classified by nonmodifiable factors (eg, race, family history, or SES) is a better strategy for obtaining adherence. Tailored solutions should use nonmodifiable factors to develop heterogeneous profiles of individuals to determine appropriate and congruent strategies to effect positive behavior change and maintain adherence to such behaviors.

##### Sex

Primary prevention and management of chronic health conditions (ie, medication and lifestyle behaviors adherence) vary by sex, where men and women display different levels of adherence across a wide variety of health behaviors and health conditions. Cross-sectional and longitudinal analyses of sex differences in medication adherence show consistently that women compared with men had less medication intensity, adherence to medications, and guidance and recommendations on drug use, especially among individuals with a chronic health condition [19,20]. Several studies indicate that men with HIV or diabetes were more adherent to physical activity recommendations and medication regimens compared with their female counterparts, partly due to greater levels of self-efficacy [21,22]. Conversely, other studies indicate that women typically reported higher health literacy levels, but lower on psychosocial determinants such as depression and social support, which are critical to medical adherence.

##### Age

Age is a significant predictor of adherence to primary prevention and management of chronic health conditions. Adherence levels across age groups are mixed, as some studies have shown that younger and older individuals display varying levels of adherence. In some studies, older adults reported greater levels of adherence, whereas in other studies, younger individuals had greater levels of adherence, depending on several factors such as the type of adherence behavior, health literacy, cultural beliefs, the personality of the individual, physical and cognitive impairment, self-perceptions of susceptibility, vulnerability and/or importance of health condition, and nature of chronic health conditions [23,24]. Studies also show that younger individuals compared with older individuals are more likely to be early adapters of certain treatments, while older individuals are more likely to demonstrate prolonged adherence. Generally, in web-based interventions, younger individuals have higher rates of intervention uptake compared with older individuals, and older individuals have higher levels of prolonging adherence compared with younger individuals [25].

##### Race/Ethnicity

Several studies have indicated that certain racial/ethnic groups have demonstrated varying levels of adherence to primary prevention and management behaviors of chronic health conditions. In a cross-sectional study of Medicare recipients living in Chicago, Gerber, Young, Ahsan, and Shoou-Yih found that race influenced medication adherence, with elderly African American patients being less likely to follow physician instructions than their white counterparts, even after adjusting for potential confounding effects of depression, sociodemographic factors, health literacy, and social support [26]. Race may also affect adherence to healthy lifestyle behaviors such as physical activity and dietary guidelines. Blacks and Mexican-Americans were less compliant with national physical activity/exercise recommendations of 150 min of moderate activity per week and reported higher levels of inactivity relative to white participants [27]. It should be noted that racial/ethnic differences in adherence are often confounded by several factors, such as access to health or medical resources, culture, income/SES, age, education level, language concordance, health literacy, and disability status [26-33].

##### Socioeconomic Status

Low SES (a combination of an individual’s education and income) and under-resourced communities typically affect adherence because they impede an individual’s ability to easily access quality and value-based health care. In addition, individuals in low-income and under-resourced neighborhoods are less likely to engage in healthy lifestyle behaviors such as healthy diet, physical activity/exercise, adequate sleep, and low stress [34,35]. Many low-income communities do not have easy access to gyms/fitness centers, doctors’ offices, healthy food grocers, parks and greenspaces, or pharmacies [36-41]. The lack of reliable transportation to commute to other neighborhoods that might have access to these health-promoting resources further compounds the dire nature of this situation [36,42]. In addition, low-income individuals with low health literacy may find it extremely difficult to adhere to health recommendations because they may not have the wherewithal and knowledge to advocate on their behalf to access quality and value-based health care [43].

#### Modifiable Determinants of Adherence

Compared with nonmodifiable determinants of adherence, modifiable determinants can be altered to increase adherence to primary prevention and management of chronic health conditions strategies. Some notable modifiable determinants of adherence include social support, motivation, emotional status, stress, health literacy, forgetfulness, health system, patient-provider communication, cost of health services, and health coverage and insurance.

##### Social Support

Social support is considered one of the most predictive modifiable factors in adherence to primary prevention and management of chronic health conditions. In a randomized control trial of 269 men and women aged 50 to 65 years, Oka, King, and Young found that support from friends, family, and exercise staff were the strongest predictors of adherence to maintaining physical activity and exercise regimen after 1 year [44]. Social support, via social media and network channels, is also integral in mHealth programs that attempt to increase adherence to primary prevention and management of chronic conditions such as physical activity, healthy diet, coping and stress reduction, and medication adherence [45,46].

##### Motivation

Motivation, which is the intrinsic or extrinsic driving force for initiating and maintaining goal-oriented behaviors, is another significant predictor of adherence to primary prevention and management of chronic health conditions. Motivation is key in initial uptake, adaptation, and maintenance of adherence behavior [47]. In chronic health conditions, motivation has proved effective in initial uptake and adaptation of health behaviors, but has shown mixed results in maintaining adherence behaviors, such as weight loss. Another challenge in optimizing motivation to increase adherence is to identify appropriate motivators for individuals across different contexts. For example, intended health benefits of adherence have proved to be insufficient in motivating individuals to adhere to healthy lifestyle prevention strategies in reducing the risk of obesity and cardiometabolic conditions [48]. Instead, other motivators such as incentives have been used successfully in mental health, home-based health monitoring, and exercise [49-51].

##### Emotional Status and Stress

Emotional status (depressive and anxiety symptoms) and stress can affect primary prevention and management of chronic health conditions, such as physical activity/exercise, sleep, and diet. Luyster, Hughes, and Gunstad conducted a cross-sectional study of 88 patients with heart failure and found that patients with symptoms of anxiety and depression are less likely to comply with their doctor-provided diet [52]. Perceived level of stress is another barrier, though more applicable to behavioral than medication adherence. In a cross-sectional study aimed at identifying barriers to exercise among women aged 40 years and older (745 African American, 660 Hispanic, 738 Native American/Native Alaskan, and 769 white), researchers found that individuals who reported feeling *too tired* for physical activity generally did so when they had a stressful day of work [53]. Stress has also been linked to poor adherence to healthy diets. Zellner and colleagues conducted an experiment to test the effect of stress on food choices among 34 female undergraduate students [54]. Participants were placed in a room with four different food choices (chips, peanuts, grapes, and M&Ms) and were asked to solve several problems with varying difficulty. The findings indicated that participants under more stress were more likely to eat M&Ms than healthier food options such as grapes. This concept known as emotional eating, due to high levels of distress, is associated with increased intake of high-calorie, low-nutrient foods, leading to weight gain and poor health outcomes [55-57]. Generally, these associations are not putative and are often mediated or confounded by nonmodifiable factors such as sex/gender. In a study that investigated self-management behavior among individuals with diabetes, men with lower levels of depression and anxiety displayed greater levels of self-management and adjustment to disease-related challenges relative to their female counterparts [58].

##### Health Literacy

Over 90 million Americans report inadequate literacy about healthy behaviors and lifestyle. Inadequate health literacy may compromise adequate comprehension of primary prevention and management of chronic health conditions. Muir et al [59] found that individuals with higher levels of health literacy are more likely to adhere to health recommendations than those with lower levels of health literacy. Gazmararian et al [60] also found that individuals with inadequate health literacy had greater odds of low refill medication adherence than those with adequate health literacy. Health literacy may also be affected by nonmodifiable and modifiable determinants of adherence behaviors, such as age, education level, cognitive impairment, dosing frequency, and patient-related health concerns [23,61-64].

##### Cognitive Factors (Memory and Information Processing)

Memory impairment and difficulty in processing health information and instructions are associated with poor adherence to primary prevention and management of chronic health conditions. Patients forget 40% to 80% of their health information and instructions given to them by their health care providers, thus compromising their ability to prevent or manage their risk for chronic health conditions [65]. In one study, impairment to prospective memory accounted for 24% of nonadherence to primary prevention and management of chronic health conditions [66]. Prospective memory is defined as an individual’s ability to remember to do something at a later time and is considered one of the strongest predictors of nonadherence [67-69]. To address memory-related nonadherence, providing cues and reminder alerts have helped individuals successfully achieve adherence to primary prevention and management of chronic health conditions [69,70].

##### Health Systems

Value-based health care (the ability to access quality and affordable health care) and navigating the complex and inaccessible health system are two system-level barriers that impede adherence to primary prevention and management of chronic health conditions [71]. Expensive health care systems, navigating complex health insurance and payer systems, limited operation hours of health facilities, and difficulty in navigating the complex health system are significant health system barriers to adherence [71]. Poor and inadequate patient-provider communication has proved to be another significant barrier to primary prevention and management of chronic health conditions. In a study of 5929 patients across 13 different hospital systems, patients with inadequate health literacy reported poorer patient-centered communication [72]. Poor patient-centered communication may be a derivative of the current payment structure in health care, which limits and disincentivizes the length of time patients have with providers. Cost and financial stress are additional health system barriers to adherence [73-76]. For example, in a systematic literature review, higher out-of-pocket medical costs were associated with a decrease in medication adherence [77]. Inefficiencies in the delivery of health care services, especially interdepartmental care coordination, are another significant health system barrier to adherence. The World Health Organization in a systematic review reports that poor provider communication about follow-up plans (eg, discharge and continuation plan), side effects of treatment, treatment journey and trajectory with patients and other providers (clinicians and pharmacists), poor information technology infrastructure, and poor multidisciplinary treatment team infrastructure make it difficult for patients to adhere to recommended treatment and medical advice [9].

### Overcoming Nonmodifiable and Modifiable Barriers to Health-Related Adherence

**Figure 1:**
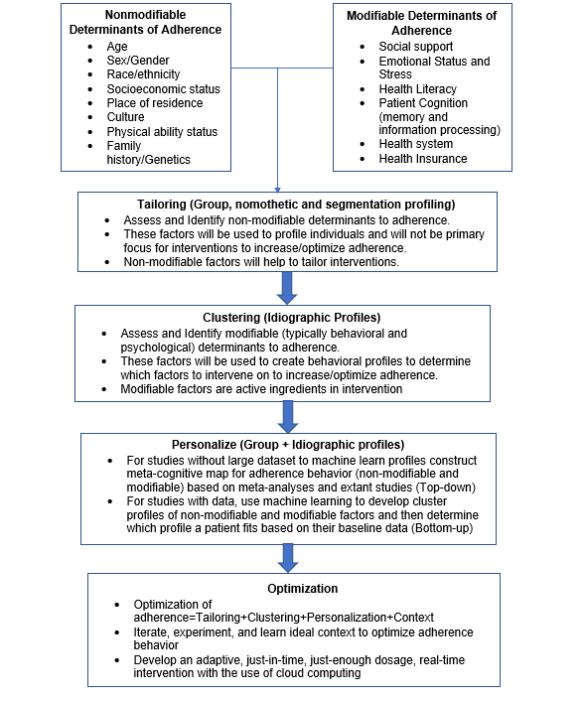
Workflow and framework to incorporate nonmodifiable and modifiable factors to improve and optimize health-related adherence behavior.

To overcome nonmodifiable and modifiable barriers of health-related adherence behaviors requires solutions that are both nomothetic/one-size-fits-all and idiographic/personalized. Such solutions must embrace a pantheoretical approach, one that incorporates nomothetic and idiographic approaches to engender precise, personalized, and optimized (contextualized) solutions to increase adherence to primary prevention and management of chronic health conditions. Nomothetic approaches are characterized as group-based or tailored interventions that apply to all and are generally based on nonmodifiable factors. However, idiographic approaches are characterized as precise and personalized interventions generally based on modifiable factors (Figure 1).

The pantheoretical approach consists of four major processes: tailoring, clustering, personalization, and optimization (Figure 1). Traditionally, tailoring is considered to be the act of customizing treatments for certain groups, based on age, race/ethnicity, and location (eg, urban/rural). However, in the pantheoretical approach, tailoring entails identifying nonmodifiable determinants of adherence behaviors and creating educational content based on profiles of nonmodifiable factors (eg, a health education program targeting black women or Hispanic men). Tailored approaches have proved effective in obtaining adherence across a wide variety of health outcomes [78,79].

Clustering, the second phase in the pantheoretical approach, consists of identifying modifiable determinants of adherence (generally behavioral or psychological), creating behavioral profiles from these nonmodifiable factors, identifying which factor(s) should be modified to increase adherence for an individual, and providing information/messages to address specific modifiable factor(s) responsible for nonadherence. The factors that are intervened upon are considered active ingredients in behavior change.

**Figure 2:**
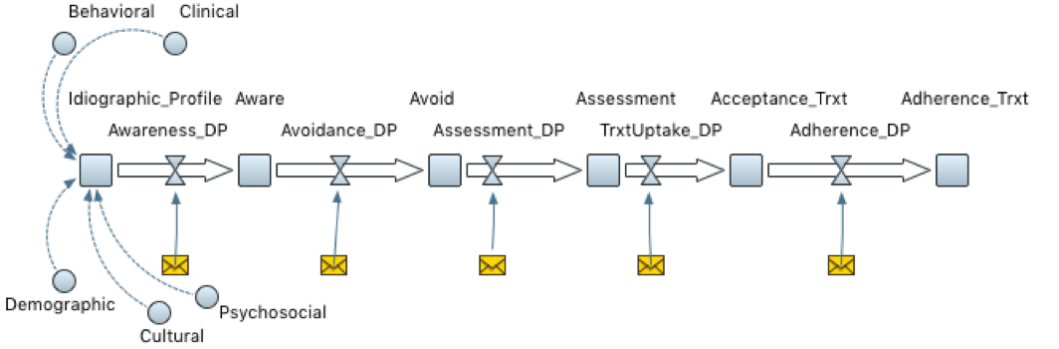
Representation of idiographic profile, meta-cognitive/mind map and care journey for an individual to increase adherence. The care continuum journey map from awareness, avoidance, assessment, acceptance, and adherence. The envelope icon represents personalized messages delivered at critical decision points to individuals to optimize acceptance and adherence treatment at each juncture of care continuum journey map.

Personalization, which is the third phase, consists of developing a meta-cognitive/mind map of barriers and facilitators of adherence (Figure 2) [80,81]. This meta-cognitive/mind map can be developed from qualitative and/or quantitative data, specifically from likelihood estimates from either trials, prospective studies, or meta-analyses. The first step in developing the meta-cognitive/mind map is to draw the conceptual model with all factors and how they are related to each other via arrows. The second step entails parameterizing each relationship. Typically, parameterization involves affixing weights based on the relative importance of each factor and creating quantitative equations that represent each relationship among factors in the meta-cognitive/mind map. If data are not available, the meta-cognitive/mind map can be derived from iterative consensus building (generally through DELPHI-like focus groups) and affixing numerical weights (representing relative importance of factors) and defining relationships among factors through hypothesized mathematical formulas based on extant research. It is highly recommended that simulation modeling software tools are used to test, validate, and calibrate these models. Meta-cognitive/mind map models can facilitate the initial development of an algorithm that can be utilized in interventions.

Optimization, the fourth phase, is the amalgamation of a tailored, clustered, and personalized intervention. A fully optimized intervention is adaptive, provides information, and messages just-in-time with an appropriate dosage at the right time in the right context [82]. To achieve complete optimization, several iterations, experiments, and fine-tuning are needed.

### Developing a Fully Personalized and Optimized Intervention

Personalization is defined as the incorporation and analysis of personal, behavioral, and real-time data to create highly contextual, adaptive, and automated messaging and curriculum (videos, text, and the literature) relevant to the participant’s emerging needs that can maximize behavior change. It is widely used in market research and is successful in enhancing engagement, experience, acceptance, and adherence to product recommendations. We argue that personalization is an effective strategy for changing and maintaining health behavior. However, to develop and achieve evidence-based personalization and optimization strategies, four critical steps must be taken.

#### Step 1

Develop a detailed idiographic profile represented as a meta-cognitive/mind map (Figure 2, idiographic profile and care journey of chronic disease) using an individual’s behavioral (behaviors that impact health and adherence to healthy lifestyle and wellbeing, such as sleep, physical activity, diet, and stress), demographic (eg, age, sex, and race/ethnicity), psychosocial (neighborhood and household context), clinical (any medical diagnosis or risk of disease), and cultural (attitudes, beliefs, and cognitions about health and wellness) data. Idiographic profiles consist of modifiable and nonmodifiable determinants of the 6 As of the chronic disease care journey: awareness, avoidance, access, assessment, acceptance, and adherence. The goal of a personalized approach is to target modifiable determinants (such as attitudes, behaviors, beliefs, and cognitions) to increase the likelihood of awareness, avoidance, access, assessment, acceptance, and adherence behaviors.

#### Step 2

Develop a generic health care-continuum journey map to achieve adherence for a specific health condition via focus groups from multiple stakeholders. The care journey map should identify critical bottlenecks and decision points (moments where participants are likely to relapse or adhere) in achieving treatment adherence as well as potential modifiable factors (intentions, behaviors, and motivations) for each bottleneck and decision point. As key modifiable factors for each bottleneck and decision point are idiosyncratic, developers should capture as much data on a wide range of potential modifiable factors before intervention deployment.

#### Step 3

Develop and train a prediction algorithm that can identify critical decision points (vulnerable and opportune times to adhere) based on the individual’s idiographic profile and care continuum journey map. This is best achieved by 1 of 2 strategies: (1) an *a priori* approach (profiling the individual via baseline data collection and determining if their idiographic profile fits a previously validated profile) and (2) *a posteriori* approach (developing an idiographic profile via baseline data or a phase-in stage where the individual is observed without being exposed to any intervention). To validate decision points, it is important to capture the input of patients in real time via responses to queries from ecological momentary assessments to maximize the timing, frequency, and duration of the intervention [83,84].

#### Step 4

After validation of decision points and bottle necks, the care journey must undergo further refinement. Beyond the initial stages of intervention exposure, the idiographic profile of each individual must be constantly updated to reflect any changes in their profile. To do so, we will capture in real time, participants’ responses to ecological momentary assessments to maximize timing, frequency, and duration of intervention exposure over a prolonged period. These data will be consistently ingested, stored, and analyzed through cloud computing. Insights obtained from cloud computing algorithms will trigger the delivery of adaptive and personalized behavioral interventions to sustain healthy behaviors. In summary, the proposed personalized models will amalgamate patient-level factors with real-time changes to create dynamic idiographic profiles, enabling the delivery of just-in-time messages that are responsive to real-time context.

### Implementing and Testing the Pantheoretical Approach to Behavior Change Through Mobile Health Technology

To successfully achieve all the components of the pantheoretical approach (tailoring, clustering, personalization, and optimization), a method that is accessible, portable, and nimble (easily modifiable and adaptable), such as mHealth technology, is needed. Embedding the pantheoretical framework in mHealth solutions can mitigate barriers and help optimize facilitators of adherence to primary prevention and management of chronic health conditions. A mHealth solution is an ideal platform and medium to achieve and sustain health behavior change through personalized and optimized strategies.

#### How Can Mobile Health Help?

Despite the proliferation of mHealth solutions and the high uptake and short-term use of such solutions, prolonged use is low and compromises long-term adherence. To engender long-term use of mHealth, it is important to address modifiable and nonmodifiable barriers. Although mHealth solutions cannot fully address the inhibitory effects of nonmodifiable barriers to adherence, they offer real and novel strategies to overcome and circumvent modifiable barriers. Some of these barriers are poor engagement, lack of motivation, limited support to achieve healthy behavior change across different contexts, inadequate health literacy, and limited access to evidence-based strategies to support healthy behavior change in real time.

One major patient-level modifiable barrier is lack of motivation, as patients may find it difficult to complete recommended tasks to achieve healthy behaviors and lifestyle. mHealth tools can modify motivation by providing timely motivators (eg, rewards, conditioned stimuli, and reinforcers) and information that may activate intrinsic beliefs and attitudes and thus induce positive behavior change. One system-level modifiable barrier is access to evidence-based strategies to support healthy behavior changes in real time. Unlike a health care provider, mHealth solutions can provide ubiquitous support (eg, out of office) to patients via mobile devices. Mobile solutions can be easily integrated into a patient’s daily routine to increase adherence to medical treatment and advice and primary prevention and management of chronic health conditions.

 First, mHealth solutions can make adherence easier and more user-friendly for patients by reducing the cognitive demand and fatigue required for optimal adherence. Specifically, mHealth solutions can optimize prospective memory and information processing, improve health literacy, reduce recall bias on health behaviors and lifestyle practices, provide social support through networks of patients with similar conditions, and facilitate effective and timely patient-provider communications. Second, mHealth solutions can also address psychosocial and health system barriers to adherence by reducing the cost of health care and increasing access to specialized and expensive health care providers. Third, mHealth solutions also allow patients to monitor their health on their own time, and access and communicate with health care providers at convenient times.

Health care providers also benefit from the use of mHealth solutions. Since providers are heavily reliant on subjective and sometimes unreliable patient reporting, an mHealth solution would allow providers to continuously monitor patient performance and adherence, reducing the likelihood of recall bias, and increasing the possibility of more personalized and adaptive interventions. mHealth solutions allow providers to be more accessible to their patients with less effort to provide real-time support through several engineering technologies, such as push-notification reminders and alerts to comply with recommended treatments, automated bots with artificial intelligence knowledge banks that can provide dynamic information to patients based on their unique conditions, and effective strategies and tips to optimize medication and healthy behavior/lifestyle adherence through the internet of things⸺things/devices that independently or jointly with other things/devices collect, store, and process data via digital or sensor-based technology, for example, wearable sensors.

The development of such a health service delivery system requires novel engineering and a paradigmatic shift in practicing medicine from a warehouse, a one-size-fits-all approach to a personalized and optimized system. An mHealth solution rooted in a pantheoretical framework (Figure 1) can revolutionize the delivery of health services from a one-size-fits-all to a personalized and optimized approach. A personalized and optimized approach can optimize adherence to prevention strategies and management of chronic health conditions, which will go at the patient’s pace, thus optimizing the likelihood of adhering to recommended medical treatment and advice, making health behavior goals more achievable.

#### Who Will Benefit From a Personalized and Optimized Mobile Health Solution?

Despite the potential benefits and successes of a personalized and optimized mHealth solution for adherence, there are some people for whom this strategy may not work. Therefore, it is important to identify those patients who are ideal candidates. There are two strategies that can be employed to determine a patient’s candidacy. The first approach utilizes an *a priori* strategy to generate a behavioral profile that includes the patient’s likelihood of being engaged, ready to participate, and adhere to an mHealth solution. The second strategy utilizes a trial-and-error run-in phase. This strategy exposes patients to the mHealth solution and monitors their level of engagement, participation, and adherence, and if after a certain period, the patient is not responsive, then it is likely that they may not be a good candidate. However, if it is critical that the patient participates in this mHealth solution, it is highly recommended to enroll the patient in a pretreatment preparatory training, which will help them be more responsive to an mHealth solution. For example, individuals above 65 years (older adults), with a chronic health condition, low-income background, racial/ethnic minority background, or inadequate health literacy are traditionally not ideal candidates for an mHealth solution [85]. Unfortunately, these are the people who need a personalized and optimized mHealth solution most. Therefore, pretreatment preparatory training would entail identifying an individual’s unique barriers to engagement with mHealth solutions and creating a graduated and sequential curriculum that involves education, simulated, and real-world use of mHealth solutions.

#### Limitations to Mobile Health Solutions: Technical, Psychosocial, and Financial Barriers

Technical, psychosocial, and financial factors that affect the adoption and prolonged use of mHealth are often overlooked yet critical barriers. Users’ reluctance to embrace mHealth solutions may include fear of technology, inability to purchase, download, and navigate mHealth and digital apps and solutions, and overly stimulating and busy interfaces making following instructions and user experience and engagement more challenging. These barriers might explain why in general one-third of individuals stop using mobile and digital solutions within 6 months of download [86].

Cost, access, privacy concerns, user satisfaction, technology literacy, and proficiency are barriers to mHealth use. Despite the fact that 1 in 5 Americans use an mHealth solution, prolonged use depends on user satisfaction, learnability, efficiency, errors, and memorability [83,84]. Data privacy and security may be other areas of concern, particularly for shared personal health information [87]. Barriers to mHealth adoption may also be due to cost, given the moderate to high-income user base [84]. The cost of a mobile device and monthly telecommunication service charge (the traditional model in the United States) serve as two major barriers. Finally, many mHealth solutions require the purchase of expensive wearable or fitness tracking devices for real-time tracking and sophisticated data visualization of user performance. Individuals are often priced out of mHealth solutions, thus giving rise to health technology inequities, where only upper- and middle-class individuals will likely benefit. Despite these limitations, the proliferation of cheaper and more affordable mobile devices as well as their use in adjunctive services provided by health care facilities and payers in comprehensive prevention health programs serve as silver linings.

### Conclusions

There are several factors that affect an individual’s adherence to health recommendations. As described above, these factors can be categorized into two categories: nonmodifiable and modifiable factors. Nonmodifiable factors are difficult to change and influence; thus, they may not provide the best opportunities to affect behavior change that leads to long-term adherence to primary prevention and management of chronic health conditions. Therefore, we argue that addressing modifiable determinants such as social support, health literacy, user motivation, emotional status, cognition (memory and information processing), and healthcare systems may provide better opportunities to affect behavior change and long-term adherence to health behaviors. We further argue that a mHealth solution may be a viable approach to address modifiable barriers and optimize adherence, while taking into consideration nonmodifiable factors, which serve to tailor, cluster/profile, personalize, and optimize interventions/strategies to obtain adherence, the pantheoretical approach.

Although mHealth solutions can be ideal for successful achievement and maintenance of adherence behaviors, they can also exacerbate barriers and thus compromise adherence. For example, low-income individuals who cannot afford mHealth solutions may be prohibited from accessing mHealth solutions, thus increasing the likelihood of nonadherence and unhealthy behaviors. In addition, the low rates of prolonged use of mHealth solutions is another critical barrier that must be addressed if it is to be used for long-term adherence. We argue that to maximize the full potential of mHealth solutions to obtain and maintain adherence, developers need to create more engaging, personalized, tailored, and multidimensional solutions (those that take into consideration the role of nonmodifiable and modifiable determinants on adherence) to achieve long-term adherence.

## Abbreviations

mHealth: mobile health
SES: socioeconomic status
